# Predictive value of preoperative platelet-to-albumin ratio and apolipoprotein B-to-apolipoprotein A1 ratio for osteosarcoma in children and adolescents: a retrospective study of 118 cases

**DOI:** 10.1186/s12885-022-09223-x

**Published:** 2022-01-27

**Authors:** Cong Ma, Ruizhen Li, Ronghui Yu, Jingjing Guo, Jianyun Xu, Xuhui Yuan, Jianfeng Guo

**Affiliations:** 1 Department of Orthopaedics, Tongji Hospital, Tongji Medical College, Huazhong University of Science and Technology, Wuhan, 430030 Hubei China; 2 Department of Oncology, West China Hospital, West China Medical College, Sichuan University, Chengdu, 610041 Sichuan China; 3 Department of Orthopaedics, First Affiliated Hospital of Nanchang University, Nanchang, 330006 Jiangxi China; 4 Department of Hematology, Union Hospital, Tongji Medical College, Huazhong University of Science and Technology, Wuhan, 430022 Hubei China

**Keywords:** Platelet-to-albumin ratio, Apolipoprotein B-to-apolipoprotein A1 ratio, Osteosarcoma, Children and adolescents, Prognosis

## Abstract

**Background:**

This retrospective study investigated biomarkers that can reflect coagulation, inflammation, and lipid abnormalities: platelet-to-albumin ratio (PAR), platelet-to lymphocyte ratio (PLR), low-density lipoprotein cholesterol to high-density lipoprotein cholesterol ratio (LDL-C/HDL-C), apolipoprotein B-to-apolipoprotein ratio (ApoB/ApoA1) whether may be viable prognostic predictors in children and adolescents with osteosarcoma.

**Methods:**

The retrospective review has enrolled a total of 118 children and adolescent patients diagnosed with osteosarcoma. Analyses with a receiver operating characteristic (ROC) curve were performed to evaluate the optimal cut-off values and to compare the area under curves (AUC). Kaplan–Meier curves were used to visualize survival outcome and a Cox proportional hazards model were used to confirm independent prognostic factors.

**Results:**

Osteosarcoma patients in high PAR group (> 4.41) and high ApoB/ApoA1 group (> 0.82) experienced significantly shorter overall survival compared with those in low PAR group (≤ 4.41) and low ApoB/ApoA1 group (≤ 0.82). In univariate and multivariable analyses, preoperative PAR and ApoB/ApoA1 were identified as independent prognostic factors for OS in children and adolescents with osteosarcoma.

**Conclusion:**

Preoperative PAR and ApoB/ApoA1 can be used as promising predictors in children and adolescents with osteosarcoma to help clinicians recognize patients with an increased risk of poor prognosis.

## Background

Osteosarcoma is the most prevalent primary malignancy of bone [1], with an average global annual incidence of 3.1 per million for all ages [2]. Adolescence is defined as the age period from 12 (early adolescence) to 25 years old (late adolescence) [3], while osteosarcoma has the highest incidence among adolescents aged 15–19 [4]. The 5-year overall survival (OS) of patients with osteosarcoma has increased from < 20% when surgery was the only therapy before the 1980s to nearly 65% after receiving combined treatment today [5, 6]. Due to the biological characteristics of osteosarcoma and the need for systemic treatment, it is strongly recommended that close follow-up, and meanwhile an accurate prediction of prognosis is related to deciding the optimal treatment plan for individual patient [2, 7]. Enneking surgical criteria, alkaline phosphatase as well as other widely-used prognostic indicators often observe heterogeneous clinical results in the same tumor stage, indicating a lack of accuracy [8, 9]. Therefore, finding accurate, practical and easy-to-detect pre-treatment indicators to assess osteosarcoma prognosis would be of great clinical significance. However, few studies have attempted to find promising potential biomarkers for predicting the prognosis of osteosarcoma, especially in children and adolescent patients.

Previous studies have shown that tumorigenesis and progression are linked with the activation of the coagulation system [10, 11], inflammation [12], and abnormalities in serum lipids and lipoproteins [13]. Platelets (PLT) are essential components of hemostasis. Recent years PLT have also been reported to be involved in the progression and metastasis of tumor cells by affecting coagulation, promoting inflammatory response and angiogenesis [14–16]. Albumin (ALB) is the most abundant plasma protein synthesized by the liver, with additional functions related to anticoagulation and anti-inflammatory, thereby inhibiting the growth of tumor cells [17, 18]. Moreover, the growing evidence revealed that lymphocyte, high-density lipoprotein cholesterol (HDL-C), low-density lipoprotein cholesterol (LDL-C), apolipoprotein A1 (ApoA1), and apolipoprotein B (ApoB) that reflect inflammation and lipid abnormalities are closely related to the progression and metastasis of tumors [19–22]. In recent years, some studies have indicated that preoperative PLT to ALB ratio (PAR), PLT to lymphocyte ratio (PLR), LDL-C to HDL-C ratio (LDL-C/HDL-C), and ApoB to ApoA1 ratio (ApoB/ApoA1) were negatively correlated with the prognosis of cholangiocarcinoma [23], lung cancer [24], colorectal cancer [25], and gastric cancer [26], respectively. Whereas, there are rarely retrospective studies concerning above indicators and the prognosis of osteosarcoma, particularly in children and adolescent patients.

Herein, this retrospective study investigated the prognostic value of preoperative PAR, PLR, LDL-C/HDL-C, and ApoB/ApoA1 in children and adolescents with osteosarcoma.

## Methods

### Study population

The present study enrolled a total of 118 children and adolescent patients with osteosarcoma who had been treated at Tongji Hospital of Tongji Medical College of Huazhong University of Science and Technology, and First Affiliated Hospital of Nanchang University from April 2012 to December 2018. The conditions for patients to enter the group included: osteosarcoma diagnosed by an experienced pathologist and without any previous anti-cancer treatment. Patients having received anticoagulant therapy or infused albumin before blood collection, used drugs affecting lipid metabolism or diagnosed with other coexisting malignancies, severe inflammation or infection in the past month, familial coagulopathy, haematology or autoimmune disease associated with elevated blood lipids (diabetes, hyperlipidemia or metabolic syndrome), blood transfusion within 4 months prior to admission or with incomplete data were excluded. Demographic information and clinical and laboratory parameters of all enrolled patients were collected, evaluated and recorded after admission. The treatment protocols of all patients were formulated and performed in accordance with the guidelines of the National Comprehensive Cancer Network. The clinical records were reviewed with the informed consent of the patients or their legal guardians.

### Clinical parameters and laboratory results

The patient's baseline characteristics as well as clinical parameters and laboratory results including age, gender, tumor size, tumor site, histological type, Enneking stage, pathological fracture, neoadjuvant chemotherapy, local recurrence, metastasis, and laboratory data (PLT, ALB, lymphocyte count, HDL-C, LDL-C, ApoA1, ApoB) were collected from the electronic medical records. In order to perfect the preoperative preparation, all patients were required to quit smoking and drinking 2 weeks before the operation and switched to light meals. Pre-meal fasting blood specimens were collected within 10 days before surgery.

### Follow-up

All the patients who were discharged after treatment were followed up every 3 months for the first 2 years, and then every 6 months for the next 3–5 years, and annually thereafter. The endpoint event is the death of the patient and the censoring time of the experiment is until March 2020. OS was calculated from the date of surgery to death or the last follow-up, and was obtained mainly through hospital records or telephone surveys.

### Statistical analyses

IBM software SPSS version 23.0 (SPSS, Chicago, IL, USA) and GraphPad Prism version 7.00 (GraphPad Software, La Jolla, CA, USA) were used to perform statistical calculations. The distribution of data was utilized using the Kolmogorov–Smirnov test. Continuous variables were presented as mean ± standard deviation (SD) and discontinuous variables were expressed as median (range). Categorical variables were presented as percentages and evaluated by chi-squared test. A receiver operative characteristic (ROC) curve was calculated to determine evaluate the optimal cut-off values and to compare area under curves (AUC). Kaplan–Meier curves were used to visualize survival outcome and a Cox proportional hazards model were used to confirm independent prognostic factors. The results were pooled using two-sided *P*-value of < 0.05 for each outcome.

## Results

### Clinical characteristics of the subjects

The patient’s baseline characteristics were shown in Table 1. 118 children and adolescent patients with osteosarcoma included 72 (61.0%) men and 46 (39.0%) women. The mean age was 16.4 ± 3.9 years, and the median maximum tumor diameter was 4.8 cm. According to Enneking surgical staging criteria, patients of stages I–II and III was 85 (72.0%) and 33 (28.0%), respectively. Pathological fracture was presented in 27 patients (22.9%). Moreover, local recurrence and metastasis occurred in 25 (21.2%) and 45 (38.1%) patients, respectively. The neoadjuvant chemotherapy was given to 71 patients (60.2%).

**Table 1:** The clinical characteristics of all patients

**Characteristics**	**Patients**	%
**Age (years)**	16.4 ± 3.9	
**Gender**		
Male	72	61.0
Female	46	39.0
**Tumor size (cm)**		
≤ 5	77	65.3
> 5	41	34.7
**Tumor site**		
Extremities	85	72.0
Non-extremities	33	28.0
**Histological type**		
Well-differentiated	68	57.6
Poorly differentiated	50	42.4
**Enneking stage**		
I	49	41.5
II	36	30.5
III	33	28.0
**Pathological fracture**		
Yes	27	22.9
No	91	77.1
**Neoadjuvant chemotherapy**		
Yes	71	60.2
No	47	39.8
**Local recurrence**		
Yes	25	21.2
No	93	78.8
**Metastasis**		
Yes	45	38.1
No	73	61.9

Continuous variables with normality were presented as mean ± standard deviation

Categorical variables were shown as percentages

### Optimal cut-off value for PAR, PLR, LDL-C/HDL-C, and ApoB/ApoA1

**Fig. 1:**
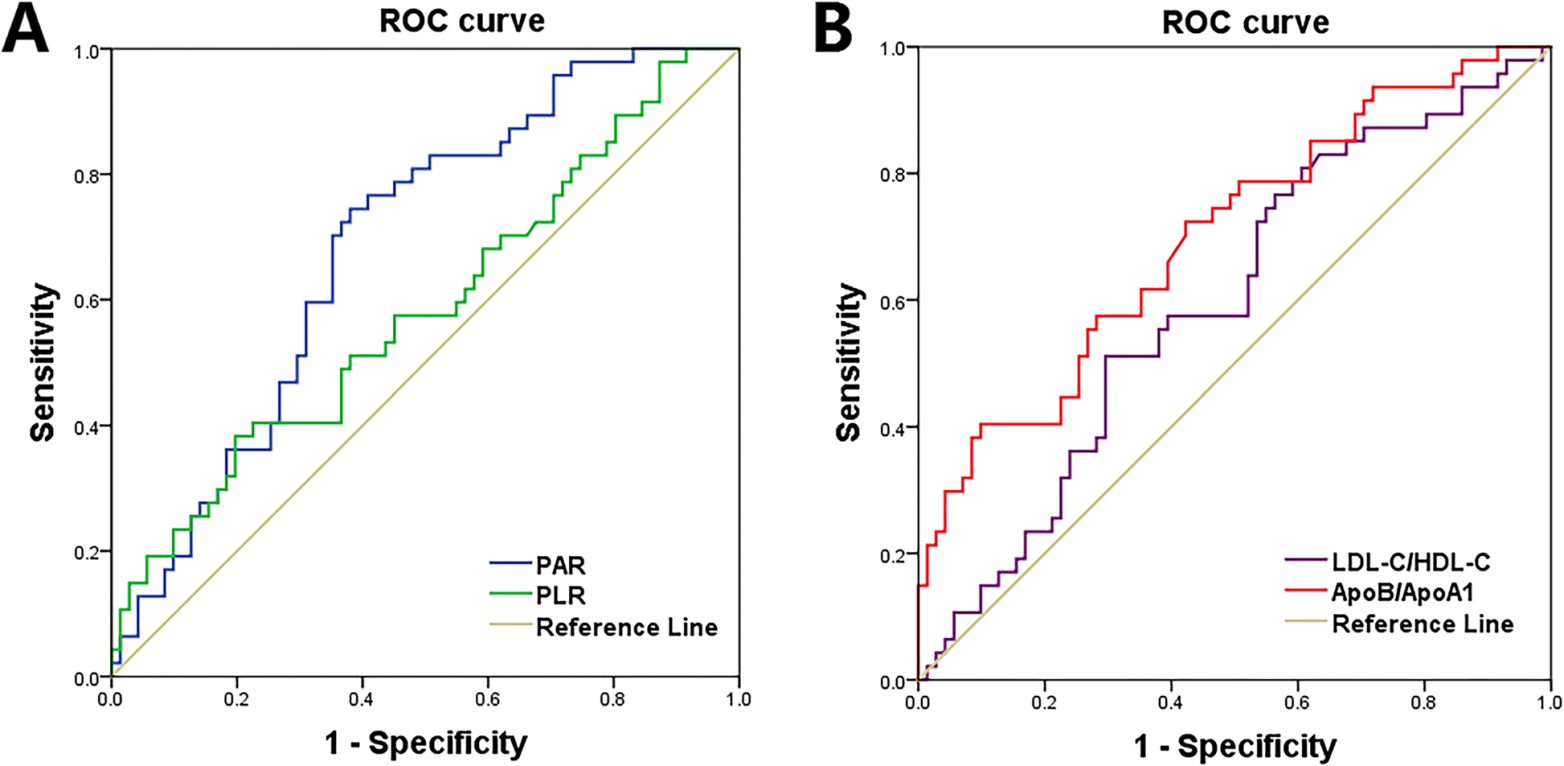
ROC curves of preoperative (**A**) PAR and PLR, and preoperative (**B**) LDL-C/HDL-C and ApoB/ApoA1 for predicting OS in children and adolescents with osteosarcoma

As shown in Table 2 and Fig. 1, ROC curve analysis was calculated for OS in children and adolescent patients with osteosarcoma. The cut-off value of joint maximum sensitivity and specificity for PAR was 4.41 (*P* = 0.001), PLR was 209.54 (*P* = 0.036), LDL-C/HDL-C was 2.04 (*P* = 0.017), and ApoB/ApoA1 was 0.82 (*P* < 0.001). According to each cut-off value, the patients were divided into high PAR or low PAR group (> 4.41 or ≤ 4.41, respectively), high PLR or low PLR group (> 209.54 or ≤ 209.54, respectively), high LDL-C/HDL-C or low LDL-C/HDL-C group (> 2.04 or ≤ 2.04, respectively), and high ApoB/ApoA1 or low ApoB/ApoA1 group (> 0.82 or ≤ 0.82, respectively).

**Table 2:** Identification of optimal cut-off values for different prognostic factors based on the ROC curve

Factor	Optimal cut-off value	Sensitivity	Specificity	Youden index	Positive predictive value	Negative predictive value	Overall accuracy rate	AUC	95% CI	*P* value
PAR	4.41	0.745	0.620	0.365	0.565	0.786	0.669	0.686	0.591–0.781	0.001
PLR	209.54	0.393	0.803	0.196	0.563	0.663	0.636	0.593	0.477–0.690	0.036
LDL-C/HDL-C	2.04	0.511	0.704	0.215	0.533	0.685	0.627	0.606	0.493–0.700	0.017
ApoB/ApoA1	0.82	0.723	0.582	0.305	0.630	0.641	0.636	0.699	0.603–0.796	< 0.001

Data were analyzed by the ROC curve. *P* < 0.05 was considered significant

### Association of clinical characteristics with preoperative PAR, PLR, LDL-C/HDL-C, and ApoB/ApoA1

Table 3 showed the relationship between PAR, PLR, LDL-C/HDL-C, ApoB/ApoA1, and clinical characteristics. The results presented that PAR was significantly associated with gender (*P* = 0.020), Enneking stage (*P* = 0.020), pathological fracture (*P* = 0.011), local recurrence (*P* = 0.008). Besides, PLR was significantly correlated with Enneking stage (*P* = 0.020). In addition, ApoB/ApoA1 was significantly related with gender (*P* = 0.027), and pathological fracture (*P* = 0.032). There was no significant relationship between preoperative high or low groups of PAR, PLR, LDL-C/HDL-C, ApoB/ApoA1, and other clinical characteristics except the above (*P* > 0.05 for all).

**Table 3:** Associations of clinical characteristics with preoperative PAR, PLR, LDL-C/HDL-C, and ApoB/ApoA1

Characteristics	PAR			PLR			LDL-C/HDL-C			ApoB/ApoA1		
	**High**	**Low**	***P* value**	**High**	**Low**	***P* value**	**High**	**Low**	***P* value**	**High**	**Low**	***P* value**
**Age (years)**			0.985			0.850			0.780			0.804
≤ 16	30	27		15	42		21	36		12	45	
> 16	32	29		17	44		24	37		14	47	
**Gender**			0.020			0.057			0.179			0.027
Male	44	28		24	48		24	48		11	61	
Female	18	28		8	38		21	25		15	31	
**Tumor size (cm)**			0.859			0.702			0.347			0.987
≤ 5	40	37		20	57		27	50		17	60	
> 5	22	19		12	29		18	23		9	32	
**Tumor site**			0.133			0.062			0.149			0.392
Extremities	41	44		19	66		29	58		17	68	
Non-extremities	21	12		13	20		16	17		9	24	
**Histological type**			0.786			0.306			0.979			0.994
Well-differentiated	35	33		16	52		26	42		15	53	
Poorly differentiated	27	23		16	34		19	31		11	39	
**Enneking stage**			0.020			0.020			0.550			0.529
I/II	39	46		18	67		31	54		20	65	
III	23	10		14	19		14	19		6	27	
**Pathological fracture**			0.011			0.187			0.095			0.032
Yes	20	7		10	17		14	13		10	17	
No	42	49		22	69		31	60		16	75	
**Neoadjuvant chemotherapy**			0.105			0.752			0.976			0.872
Yes	33	38		20	51		27	44		16	55	
No	29	18		12	35		18	29		10	37	
**Local recurrence**			0.008			0.261			0.108			0.418
Yes	19	6		9	16		13	12		7	18	
No	43	50		23	70		32	61		19	74	
**Metastasis**			0.203			0.734			0.651			0.340
Yes	27	18		13	32		16	29		12	33	
No	35	38		19	54		29	44		14	59	

Data were present with Chi-square test. *P* < 0.05 was considered significant

### Prognostic value of preoperative PAR, PLR, LDL-C/HDL-C, and ApoB/ApoA1

**Fig. 2:**
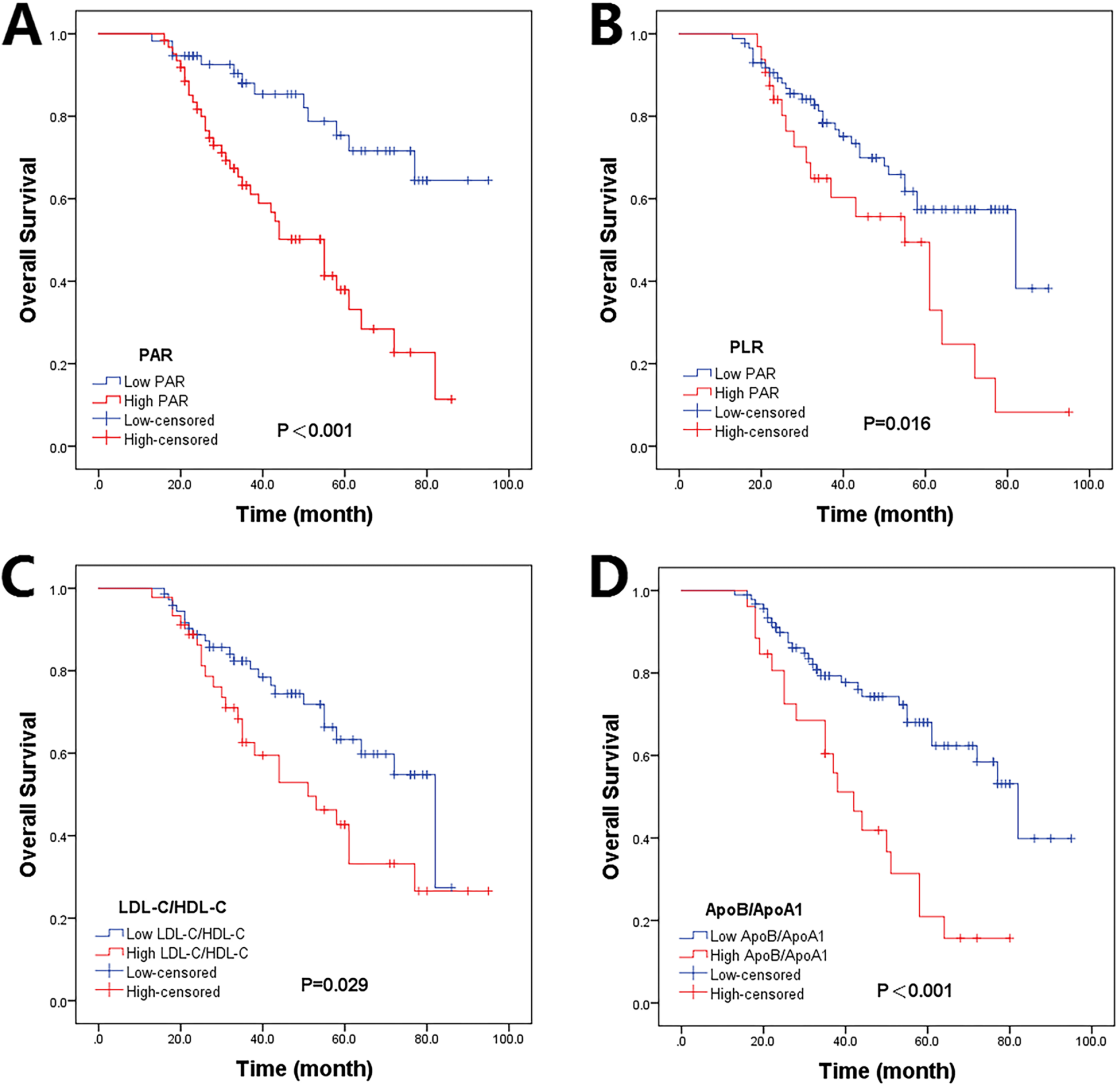
Kaplan–Meier curves for OS in children and adolescents with osteosarcoma according to (**A**) PAR, (**B**) PLR, (**C**) LDL-C/HDL-C and (**D**) ApoB/ApoA1

Kaplan–Meier curves were conducted to perform the survival analysis (Fig. 2). Compared with patients in the low groups of PAR, PLR, LDL-C/HDL-C, and ApoB/ApoA1, those in the high groups of PAR (*P* < 0.001, log-rank test; Fig. 2A), PLR (*P* = 0.016, log-rank test; Fig. 2B), LDL-C/HDL-C (*P* = 0.029, log-rank test; Fig. 2C), and ApoB/ApoA1 (*P* < 0.001, log-rank test; Fig. 2D) had shorter OS.

**Fig. 3:**
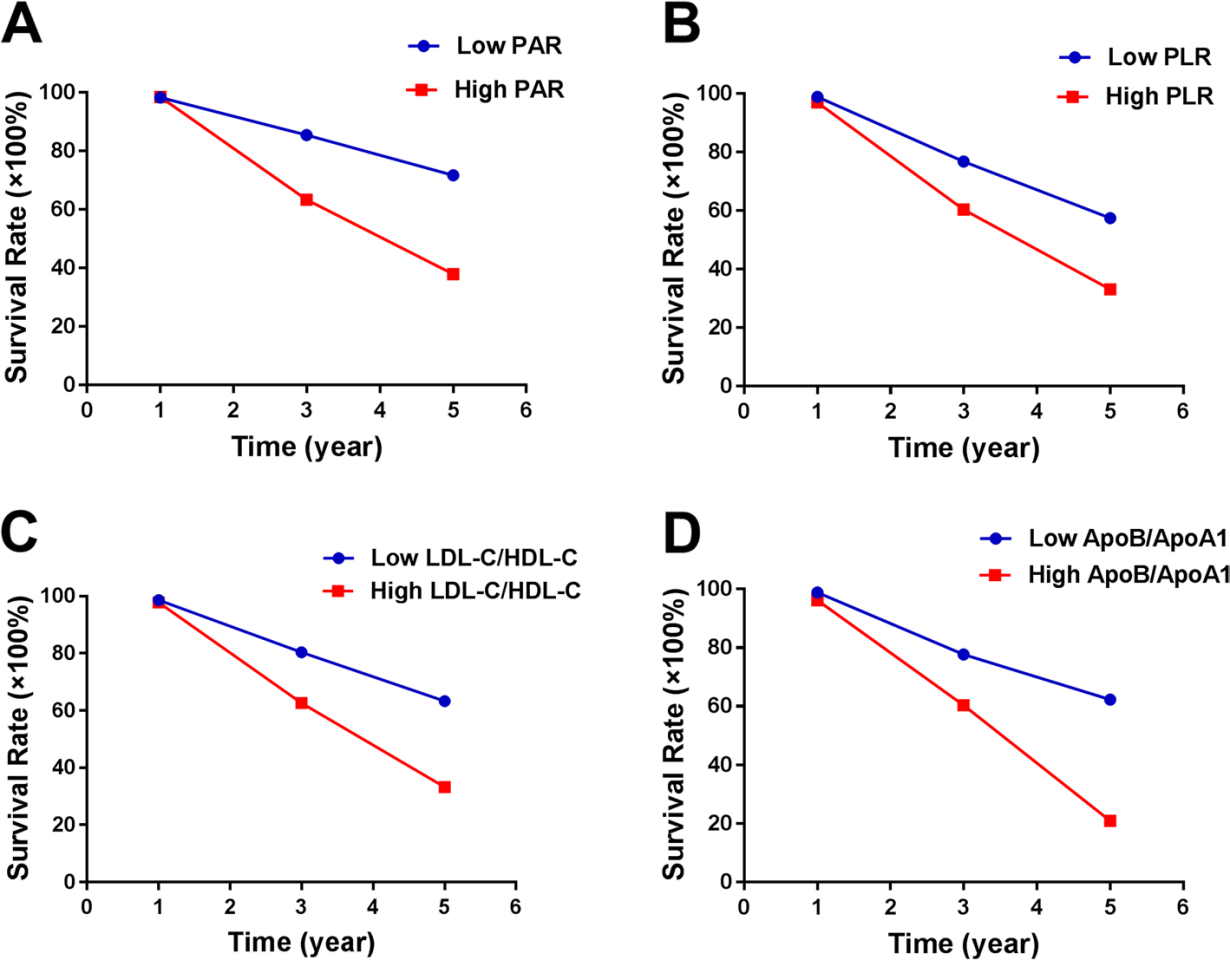
The 1-, 3- and 5-year rates of OS in all patients with osteosarcoma according to (**A**) PAR, (**B**) PLR, (**C**) LDL-C/HDL-C and (**D**) ApoB/ApoA1

The 5-year OS in low groups of PAR, PLR, LDL-C/HDL-C, and ApoB/ApoA1 were 71.6%, 57.4%, 63.3%, and 62.3%, respectively (Fig. 3). While in high groups, the 5-year OS of PAR, PLR, LDL-C/HDL-C, and ApoB/ApoA1 were 37.9%, 33.0%, 33.2% and 20.9%, respectively (Fig. 3).

**Fig. 4:**
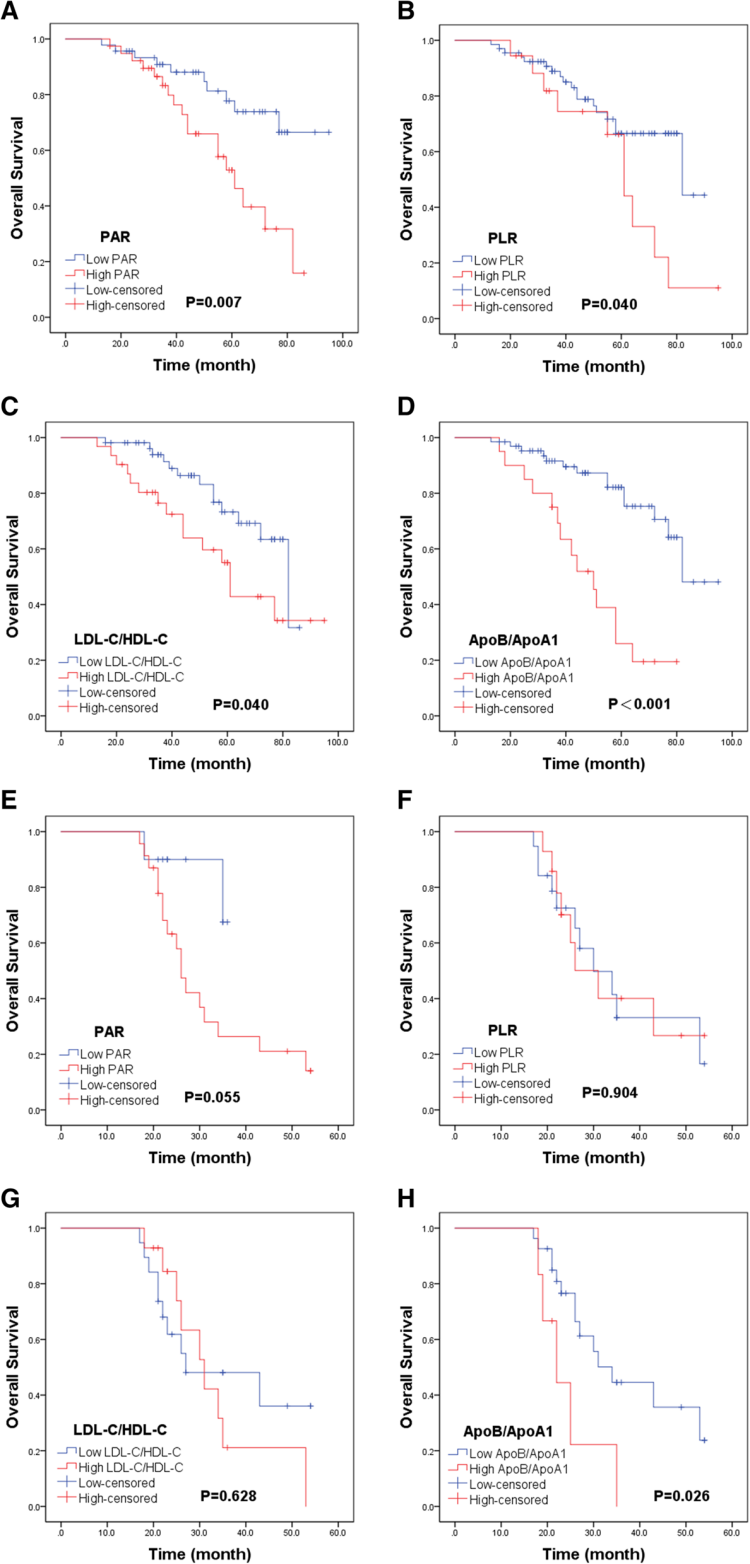
Kaplan–Meier curves of OS for PAR, PLR, LDL-C/HDL-C, and ApoB/ApoA1 in all patients stratified by Enneking stage. **A-D** Kaplan–Meier curves of OS for (**A**) PAR, (**B**) PLR, (**C**) LDL-C/HDL-C and (**D**) ApoB/ApoA1 in osteosarcoma patients with stage I-II. **E–H** Kaplan–Meier curves of OS for (**E**) PAR, (**F**) PLR, (**G**) LDL-C/HDL-C and (**H**) ApoB/ApoA1 in osteosarcoma patients with stage III

Further subgroup analysis was performed to investigate the prognostic value of PAR, PLR, LDL-C/HDL-C, and ApoB/ApoA1 in children and adolescent patients with osteosarcoma stratified by Enneking stage. The results suggested that only ApoB/ApoA1 can identify the OS differences in both stage I-II (*P* < 0.001, Fig. 4D), and III (*P* = 0.026, Fig. 4H). Additionally, Poorer OS was observed in patients with high groups of PAR (*P* = 0.007, Fig. 4A), PLR (*P* = 0.040, Fig. 4B), LDL-C/HDL-C (*P* = 0.040, Fig. 4C) in subgroup of stage I-II. However, there was no significance OS difference between the high and low groups of PAR (*P* = 0.055, Fig. 4E), PLR (*P* = 0.904, Fig. 4F), LDL-C/HDL-C (*P* = 0.628, Fig. 4G) in stage III.

Univariate and multivariate Cox proportional hazards model.

The univariate Cox proportional hazards model showed that tumor size (*P* = 0.017), Enneking stage (*P* < 0.001), pathological fracture (*P* < 0.001), local recurrence (*P* = 0.001), metastasis (*P* < 0.001), PAR (*P* < 0.001), PLR (*P* = 0.019), LDL-C/HDL-C (*P* = 0.033), and ApoB/ApoA1 (*P* < 0.001), were significantly associated with OS (Table 4). In the multivariate Cox proportional hazards model, we identified Enneking stage (*P* = 0.007), local recurrence (*P* = 0.029), metastasis (*P* < 0.001), PAR (*P* = 0.033), and ApoB/ApoA1 (*P* = 0.001) as independent prognostic factors for OS.

**Table 4:** Univariate and multivariate Cox proportional hazards model analysis for overall survival

Characteristics	Univariate analysis			Multivariate analysis		
	**HR**	**95%CI**	***P* value**	**HR**	**95%CI**	***P* value**
**Age (years)**			0.299			
≤ 16	1.000	Reference				
> 16	1.358	0.762–2.420				
**Gender**			0.910			
Male	1.000	Reference				
Female	0.983	0.734–1.317				
**Tumor size (cm)**			0.017			0.349
≤ 5	1.000	Reference		1.000	Reference	
> 5	2.006	1.130–3.563		1.408	0.688–2.883	
**Tumor site**			0.946			
Extremities	1.000	Reference				
Non-extremities	0.979	0.525–1.824				
**Histological type**			0.415			
Well-differentiated	1.000	Reference				
Poorly differentiated	1.272	0.714–2.266				
**Enneking stage**			< 0.001			0.007
I/II	1.000	Reference		1.000	Reference	
III	5.695	2.910–11.147		3.213	1.373–7.520	
**Pathological fracture**			< 0.001			0.162
Yes	1.000	Reference		1.000	Reference	
No	0.267	0.144–0.494		0.589	0.281–1.236	
**Neoadjuvant chemotherapy**			0.329			
Yes	1.000	Reference				
No	1.331	0.749–2.365				
**Local recurrence**			0.001			0.029
Yes	1.000	Reference		1.000	Reference	
No	0.384	0.213–0.693		0.420	0.193–0.917	
**Metastasis**			< 0.001			< 0.001
Yes	1.000	Reference		1.000	Reference	
No	0.286	0.158–0.516		0.252	0.123–0.518	
**PAR**			< 0.001			0.033
High	1.000	Reference		1.000	Reference	
Low	0.274	0.141–0.533		0.428	0.196–0.934	
**PLR**			0.019			0.438
High	1.000	Reference		1.000	Reference	
Low	0.494	0.274–0.892		0.766	0.390–1.504	
**LDL-C/HDL-C**			0.033			0.115
High	1.000	Reference		1.000	Reference	
Low	0.535	0.302–0.950		0.572	0.285–1.145	
**ApoB/ApoA1**			< 0.001			0.001
High	1.000	Reference		1.000	Reference	
Low	0.336	0.186–0.606		0.268	0.135–0.530	

Data were analyzed by Cox proportional hazards model. *P* < 0.05 was considered significant

## Discussion

Tumorigenesis and progression are complex processes with many contributing factors. In this retrospective study, it was found that patients with relatively high preoperative PAR (> 4.41) and high preoperative ApoB/ApoA1 (> 0.82) were associated with shorter OS. More significantly, we confirmed for the first time that preoperative PAR and ApoB/ApoA1 can be identified as independent prognostic factors for OS in children and adolescents with osteosarcoma.

The clinical value of our current findings cannot be ignored. First of all, our findings indicate that activation of the coagulation system, the enhancement of inflammatory cell response and the abnormality of blood lipid may be involved in the progression of osteosarcoma. Based on this, interventions or drugs aimed at reducing the ratio of PAR and ApoB/ApoA1 might be a potentially effective strategy to improve the prognosis of osteosarcoma. In addition, these findings may help clinicians recognize patients with increased risk of poor prognosis in osteosarcoma and establish a framework for individualized treatment of children and adolescents with osteosarcoma in the future.

Furthermore, our findings are also consistent with other previous studies. Saito et al. [23], Shirai et al. [27], and Li et al. [28] have identified that PAR can be used as an independent factor for prognosis in cholangiocarcinoma, pancreatic ductal adenocarcinoma, and hepatocellular carcinoma, respectively. Meanwhile, the results of Yang et al. [29], Ma et al. [26], and Zhang et al. [30] have shown that high preoperative ApoB/ApoA1 is a significant association with poor cancer prognosis of colorectal, gastric, and renal cell carcinoma. Whereas, as new indicators for evaluating OS, the potential mechanism of serum PAR and ApoB/ApoA1 to predict the survival outcomes of osteosarcoma remains unclear. We could only try to give a preliminary explanation by reviewing previous studies.

Falanga et al. [10, 31] reported that platelets are activated by tumor to adhere heavily to peripheral blood tumor cells, aiding proliferation by protecting the tumor cells from immune cells. As the number of activated platelets increase and aggregate, the released inflammatory factors promote angiogenesis and tumor progression [32–34]; hematogenous metastasis is promoted as tumor cell emboli are blocked in microcirculation [35–37]. The elevation of tissue factors in tumor cells, and increased release of tissue factors in platelets, further activate coagulation and fibrinolysis to promote tumor progression and metastasis [11, 38, 39]. Besides, cisplatin is a crucial chemotherapeutic drug for osteosarcoma [2], while the study of Wang et al. [40] showed that high platelet levels can activate Akt and Erk signaling, thereby saving cisplatin-induced apoptosis, thus reducing the efficacy of platinum-based therapy, leading to poor prognosis. In addition, inflammatory cytokines released from cancer cells and platelets inhibit albumin synthesis in hepatocytes [41]. Albumin can inhibit the activation and aggregation of platelets induced by histone H4, and exerts an anticoagulant effect by binding arachidonic acid [18, 42]. When the albumin level of the body decreases, these functions are weakened, and tumor progression is then promoted. Meanwhile, Low albumin level is also the manifestation of the heightened systemic inflammatory reaction and malnutrition, which also may affect the prognosis of osteosarcoma [17, 43, 44]. ApoA1, a major protein component of HDL-C and a key medium for cholesterol homeostasis, plays an anti-tumor role via affecting the immune system and inhibiting new angiogenesis [45, 46]. The decrease of ApoA1 level in circulation can transform macrophages of anti-tumor M1 phenotype into M2-macrophages that promote tumor, and reduce cytotoxic CD8^+^ T cells to enhance the inflammatory response of tumor, and promote angiogenesis and increasing the activity of MMP-9, thereby causing poor OS of tumor [47–49]. Moreover, ApoB, the main structural protein of LDL-C, can promote lipoproteins to enter the blood vessel wall, stimulate the phagocytosis of macrophages, thereby enhancing the inflammatory response to further promote tumor progression [22, 50]. Hence, the mechanisms described above may explain, at least partially, the associations between relatively high preoperative PAR and ApoB/ApoA1, and poor prognosis of children and adolescents with osteosarcoma observed in the present study.

Indeed, the current study is limited by its retrospective nature, so selection bias and inaccuracies may exist. Additionally, given that osteosarcoma is a sporadic malignant tumor, our current research is limited by the small sample size, especially concerning the patients at stage III, so that patients at this stage might not be adequate to represent the whole population. Besides, the diagnosis of osteosarcoma relies on imaging and histopathological examinations, so that it is difficult to obtain accurate progression-free survival rates for patients. Last, our findings still warrant further investigations by large sample size and multi-center studies.

## Conclusion

In summary, this study found that osteosarcoma patients with relatively high preoperative PAR and ApoB/ApoA1were associated with shorter OS. More significantly, we confirmed that preoperative PAR and ApoB/ApoA1 can be identified as independent prognostic factors for OS in children and adolescents with osteosarcoma. These findings may help clinicians recognize patients with an increased risk of poor prognosis in osteosarcoma and establish a framework for individualized treatment of children and adolescents with osteosarcoma in the future.

## Data Availability

The data that support the findings of this study are available on request from the corresponding author. The data are not publicly available due to privacy or ethical restrictions.

## Abbreviations

PLT: Platelet
ALB: Albumin
HDL-C: High-density lipoprotein cholesterol
LDL-C: Low-density lipoprotein cholesterol
ApoA1: Apolipoprotein A1
ApoB: Apolipoprotein B
PAR: Platelet-to-albumin ratio
PLR: Platelet-to-lymphocyte ratio
LDL-C/HDL-C: LDL-C to HDL-C ratio
ApoB/ApoA1: ApoB to ApoA1 ratio
OS: Overall survival
ROC: Receiver operative characteristic
AUC: Area under curve
HR: Hazard ratio
CI: Confidence interval
